# Copper Oxide-Antimony Oxide Entrapped Alginate Hydrogel as Efficient Catalyst for Selective Reduction of 2-Nitrophenol

**DOI:** 10.3390/polym14030458

**Published:** 2022-01-24

**Authors:** Sher Bahadar Khan, Esraa M. Bakhsh, Kalsoom Akhtar, Tahseen Kamal, Yan Shen, Abdullah M. Asiri

**Affiliations:** 1 Chemistry Department, Faculty of Science, King Abdulaziz University, P.O. Box 80203, Jeddah 21589, Saudi Arabia; ibakhsh@kau.edu.sa (E.M.B.); kaskhan@kau.edu.sa (K.A.); tkkhan@kau.edu.sa (T.K.); aasiri2@kau.edu.sa (A.M.A.); 2 Center of Excellence for Advanced Materials Research, King Abdulaziz University, P.O. Box 80203, Jeddah 21589, Saudi Arabia; 3 Wuhan National Laboratory for Optoelectronics, Huazhong University of Science and Technology, Luoyu Road 1037, Wuhan 430074, China; ciac_sheny@hust.edu.cn

**Keywords:** Na-alginate, hydrogel wrapped copper oxide-antimony oxide, nitrophenols, dyes, potassium ferricyanide, catalytic reduction, water treatment, environmental applications

## Abstract

Copper oxide-antimony oxide (Cu_2_O-Sb_2_O_3_) was prepared and entrapped inside Na-alginate hydrogel (Alg@Cu_2_O-Sb_2_O_3_). The developed Alg@Cu_2_O-Sb_2_O_3_ was used as catalytic reactor for the reduction of 4-nitrophenol (4-NP), 2-nitrophenol (2-NP), 2,6-dinitrophenol (2,6-DNP), methyl orange (MO), congo red (CR), acridine orange (AO), methylene blue (MB) and potassium ferricyanide (K_3_[Fe(CN)_6_]). Alg@Cu_2_O-Sb_2_O_3_ was found to be selective and more efficient for the reduction of 2-NP among all the pollutants. Therefore, 2-NP was selected for a detailed study to optimize various parameters, e.g., the catalyst amount, reducing agent concentration, 2-NP concentration and recyclability. Alg@Cu_2_O-Sb_2_O_3_ was found to be very stable and easily recyclable for the reduction of 2-NP. The Alg@Cu_2_O-Sb_2_O_3_ nanocatalyst reduced 2-NP in 1.0 min, having a rate constant of 3.8187 min^−1^.

## 1. Introduction

Environmental pollution achieved worrying levels due to the continuous release and disposal of numerous pollutants into the environment and wastewater. Many of these pollutants are dyes and nitrophenols and are unsustainable in nature, which is of particularly great concern due to environmental regulations. These pollutants have high toxicity, hazardous nature, carcinogenicity, mutagenic nature, low biodegradability, and the fact that they are harmful and dangerous to living organisms [1,2]. Currently, dyes as well as phenols are believed to be tenacious, lethal, and dangerous contaminants coming from industrial wastewater [3,4]. These pollutants exist in the wastes of agrochemical, pharmaceutical, textile, and chemical industries because several industries are accountable for throwing out various pollutants into the ecosystem through wastewater streams. Consequently, the processes related to the disposal of the above-mentioned pollutants have foremost limitations, having a very slow removal proficiency, a poor elimination rate as well as being very costly and complex, which limits its applications [5,6]. Fortunately, researchers have focused a lot on detecting significant pollutants for different water bodies, but less work has been done on the removal or conversion of toxic pollutants [1,2]. Therefore, the efficient reduction and fast degradation of these pollutants is a central concern, considering its environmental and health hazards. One way to manage these pollutants from wastewater is by performing their reduction so as to produce less toxic or useful compounds.

Various techniques are utilized regarding the removal of lethal pollutants from contaminated water [7]. However, these processes are believed to not be very useful for the complete removal of dangerous pollutants, or they require a long time. Second, the high cost also reduces their importance. Thus, easy, uncomplicated, and cheap methods are required for managing polluted wastewater [3,8]. Catalytic reduction is considered an inexpensive and good technique as it is easy, fast, and proficient. Catalytic reduction involves a very low quantity of solvent, and the time required for removal is also very short [9,10,11]. In recent times, many efforts have been made to prepare new catalysts and enhance their catalytic performance and stability as well as their selectivity regarding water pollutants [12,13,14].

Nanomaterials have eccentric and numerous applications in relation to human health, which they confer significantly to medicine and the environment. Nanomaterials demonstrate a good ability as catalysts, which is hardly possible when considering conventional systems. Nanomaterials retain a distinct shape, small size, and remarkable surface activity [15,16,17,18,19]. Tin oxide possesses a good ability and many applications, but still, it requires reformation to enhance its catalytic properties [20,21,22]. When considering many techniques, doping is the one that is used to reveal excellent properties in various areas. Dopants works to increase the surface area and alter the structure, modifying the morphology, which leads to enhanced properties of nanomaterials [23,24].

Doped metal oxides are efficient catalysts, but their utilization in powder form has several drawbacks such as aggregation, regeneration of the catalyst, recyclability and leaching. Therefore, to solve these problems, a Cu_2_O-Sb_2_O_3_ nanomaterial was wrapped inside Na-alginate hydrogel to prevent this agglomeration and to make the recycling of metal oxide nanocatalysts easy, which is the novelty of the current study. For the current study, Cu_2_O-Sb_2_O_3_ nanomaterial was prepared and checked by FESEM, EDS, XRD and FTIR. Further Cu_2_O-Sb_2_O_3_ was entrapped inside Na-alginate hydrogel. Alg@Cu_2_O-Sb_2_O_3_ was initially tested for MO, CR, ArO, MB and K_3_[Fe(CN)_6_], 4-NP, 2-NP and 2,6-DNP, and we explored the efficiency of the Alg@Cu_2_O-Sb_2_O_3_ nanocatalyst for the reduction of these pollutants. The Alg@Cu_2_O-Sb_2_O_3_ nanocatalyst achieved the greatest selectivity toward 2-NP, which underwent a reduction in 1.0 min, having a superior rate constant.

## 2. Experimental Section

### 2.1. Chemicals and Reagents

Sodium alginate (NaC_6_H_7_O_6_)_n_, copper nitrate, antimony chloride, K_3_[Fe(CN)_6_] and NaBH_4_ were obtained from Sigma Aldrich. 4-NP, 2-NP and 2,6-DNP were obtained from Fluka. MO, CR, ArO, and MB were obtained from Merck, Germany.

### 2.2. Preparation of Cu_2_O-Sb_2_O_3_ and Alg@Cu_2_O-Sb_2_O_3_

**Scheme 1:**
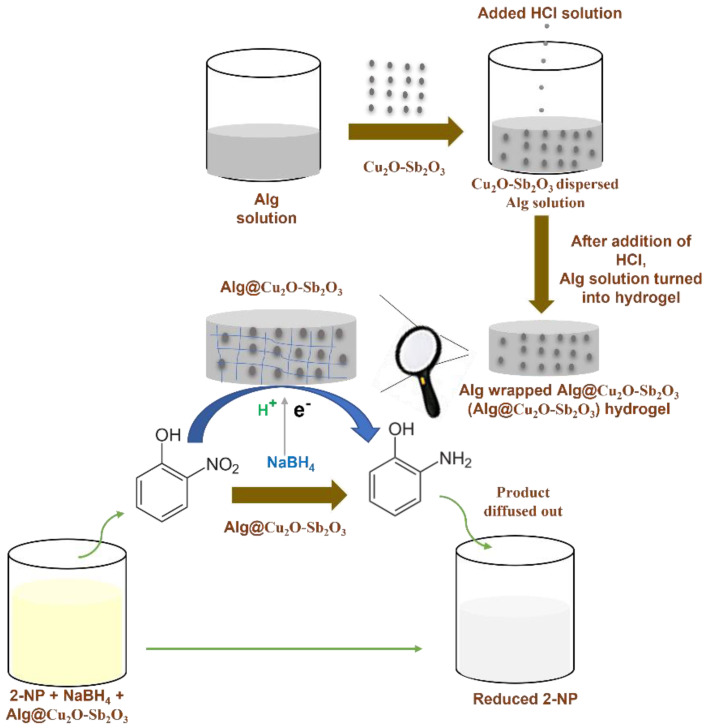
Scheme of Alg@Cu_2_O-Sb_2_O_3_ preparation and catalytic reduction of 2-NP.

0.1 M solutions of antimony chloride and copper nitrate were initially prepared and then mixed. The mixed aqueous solution was stirred continuously, and then 0.1 M NH_4_OH was added and the pH was increased to 10. The mixed solution was heated at 150.0 °C for 10 h in an oven using an autoclave. The product was then filtered. After filtration, the product was dried after washing it thoroughly and was calcined at 400.0 °C for 5 h. The preparation process of Alg@Cu_2_O-Sb_2_O_3_ and its utilization has been shown in Scheme 1. Alg@Cu_2_O-Sb_2_O_3_ composite hydrogel was readily prepared by dispersing 3, 5 and 10 mg of Cu_2_O-Sb_2_O_3_ in 1 mL of Alg individually. After that, HCl solution was added to the mixed solution. Alg was turned into hydrogel by H^+^ after adding HCl solution because of H^+^ crosslinking Alg. Alg@Cu_2_O-Sb_2_O_3_ was finally removed and washed by utilizing distilled water for the purpose of removing the unreacted H^+^ present on the top surface of the hydrogel. The samples were examined by a field emission scanning electron microscope (FESEM, INSTRUMENT JSM-7600F, Japan), energy dispersive X-ray spectrometry (EDS, Oxford instruments Inc system), powder X-ray diffraction (XRD, ARL X’tra Thermo Scientific diffractometer), and a Fourier Transform Infrared spectrometer. The catalytic reduction was examined by a UV–Vis spectrometer (Evolution 300 by Thermo Scientific).

### 2.3. Catalytic Reduction

At first, the Alg@Cu_2_O-Sb_2_O_3_ hydrogel nanocatalyst was assessed with different dyes (CR (0.07 mM), MO (0.07 mM), ArO (0.07 mM), MB (0.07 mM)), K_3_[Fe(CN)_6_] (2.0 mM) and nitrophenols (4-NP (0.13 mM), 2-NP (0.13 mM) and 2,6-DNP (0.13 mM)). The catalytic reaction of the selected dyes, K_3_[Fe(CN)_6_] and nitrophenols was performed by Alg@Cu_2_O-Sb_2_O_3_ catalyst in the presence of NaBH_4_. 2.5 mL of pollutant solution was poured in a cuvette, and its UV spectrum was checked. 0.5 mL of NaBH_4_ (0.1 M) was poured into it, followed by the addition of the Alg@Cu_2_O-Sb_2_O_3_ hydrogel nanocatalyst to the reaction system, and then the UV–vis spectrum was measured with an interval of 1.0 min. Consequently, the catalytic reactions of CR, MO, ArO, MB, K_3_[Fe(CN)_6_], 4-NP, 2-NP and 2,6-DNP were analyzed by measuring the absorbance spectra at 490 nm, 460 nm, 465 nm, 658 nm, 415 nm, 400 nm, 413 nm and 428 nm, respectively, and the percent reduction (%*R*) was measured using Equation (1):

(1) $$\%R=\frac{C_{i}-C_{e}}{C_{i}}\times 100$$

*C_i_* = initial and *C_e_* = final concentrations of pollutants.

## 3. Results and Discussion

### 3.1. Selection and Structure Interpretation of Cu_2_O-Sb_2_O_3_ and Alg@Cu_2_O-Sb_2_O_3_

In search of an efficient, stable, and economical catalyst, a huge number of materials have been investigated. Among different materials, nanomaterials containing metal oxides play a very important part as a catalyst. As a catalyst, metal oxides have displayed good catalytic activities when considering different reactions. Thus, it was thought that doped metal oxides could be an excellent selection for the reduction reaction of pollutants. Consequently, Cu_2_O-Sb_2_O_3_ and Alg@Cu_2_O-Sb_2_O_3_ were prepared and tested in a reduction reaction of pollutants and discovered to be good catalysts because the Alg@Cu_2_O-Sb_2_O_3_ nanocatalyst showed a superb catalytic activity in relation to the catalytic reduction reaction of pollutants. However, it was more efficient for the reactions involving the 2-NP reduction, and it was, therefore, suggested that the Alg@Cu_2_O-Sb_2_O_3_ nanocatalyst was a better choice for the reduction of 2-NP. Additionally, the composition, chemical structure, and morphology of Cu_2_O-Sb_2_O_3_ was evaluated by FESEM, EDS, XRD and ATR-FTIR. The wrapping of Cu_2_O-Sb_2_O_3_ inside Alg was confirmed by XRD and ATR-FTIR.

**Figure 1:**
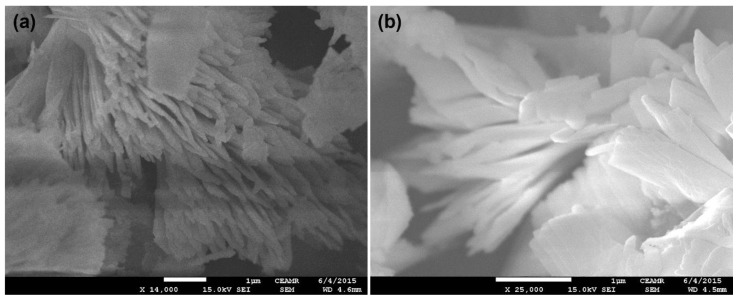
FESEM images of Cu_2_O-Sb_2_O_3_ at (**a**) ×14,000 and (**b**) ×25,000.

FESEM was utilized to check the morphology of Cu_2_O-Sb_2_O_3,_ and the FESEM images are displayed in Figure 1. The surface morphology of Cu_2_O-Sb_2_O_3_ was explicated by comparing both the low and high magnification images. The Cu_2_O-Sb_2_O_3_ images indicate that it is grown like sheets, which combine to construct a flower-shaped structure. Thus, Cu_2_O-Sb_2_O_3_ is grown in nanosheets, for which the sheet diameter was observed to be ~10 nm. The images clearly show that Cu_2_O-Sb_2_O_3_ has a flower-shaped morphology, where the flower is made of aggregated nanosheets, collectively giving a flower structure.

**Figure 2:**
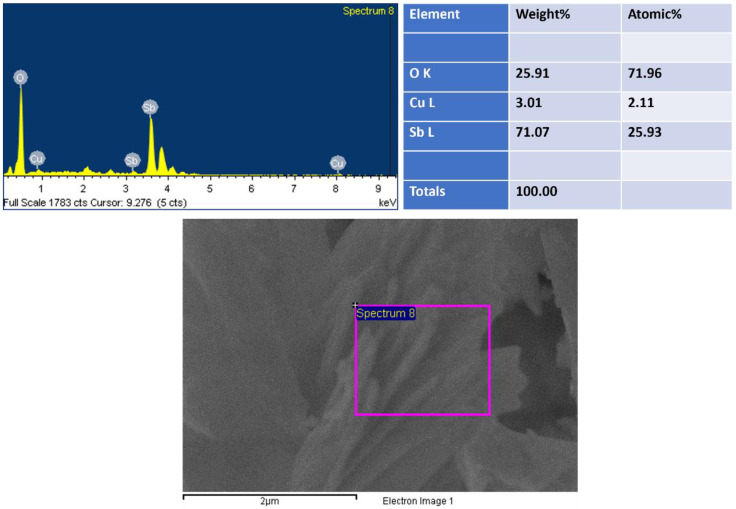
EDS of Cu_2_O-Sb_2_O_3_.

By using EDS, the compositional analysis of nanomaterial was examined, as shown in Figure 2. EDS displayed peaks related to antimony (Sb), copper (Cu) and oxygen (O), showing the existence of the corresponding elements and suggesting their existence in the prepared nanomaterial. EDS revealed 71 wt% of Sb and 3 wt% of Cu, indicating the growth of Cu_2_O-doped Sb_2_O_3_. The lack of any extra peak in EDS suggested that the synthesized Cu_2_O-Sb_2_O_3_ nanomaterial contained no impurity.

**Figure 3:**
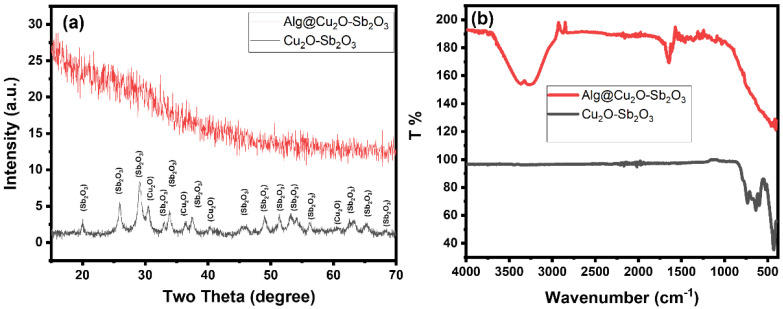
(**a**) XRD and (**b**) ATR-FTIR of Cu_2_O-Sb_2_O_3_ and Alg@Cu_2_O-Sb_2_O_3_ nanocatalysts.

The XRD patterns of the prepared material clearly reveal peaks that correspond to the crystalline phase of Cu_2_O and Sb_2_O_3_ (Figure 3a). Hence, the XRD patterns imply that the prepared material is comprised of Cu_2_O and Sb_2_O_3_ phases. Cu_2_O-Sb_2_O_3_ revealed diffraction peaks at 2θ = 20.0, 26.0, 29.3, 30.5, 33.0, 33.9, 36.4, 37.5, 40.3, 41.8, 45.8, 47.4, 49.1, 51.5, 53.2, 54.1, 56.3, 60.8, 63.2, 65.2, and 68.3°. The diffraction lines at 2θ = 30.5, 36.4 and 40.3°, which could be indexed to (110), (111), and (200), were assigned to Cu_2_O [25,26]. All other peaks are well matched with the literature and could be assigned to the Sb_2_O_3_ phase. The intensities of the Cu_2_O peaks are low in the XRD pattern, which might be due to the low quantity of Cu_2_O phase present in Cu_2_O-Sb_2_O_3_ when compared to the Sb_2_O_3_ phase. Therefore, the Sb_2_O_3_ diffraction peaks have significantly high intensities when compared to the Cu_2_O peaks. The XRD pattern suggests the possible formation of a two-phase material composed of Cu_2_O and Sb_2_O_3_ phases [24,27,28,29]. The Alg@Cu_2_O-Sb_2_O_3_ hydrogel XRD only demonstrated the pattern of the amorphous material. This peak can be attributed to the amorphous phase of Alg. The Alg@Cu_2_O-Sb_2_O_3_ pattern did not show any peak associated with the Cu_2_O-Sb_2_O_3_ phase, the reason for this being either the presence of a lower quantity of Cu_2_O-Sb_2_O_3_ in Alg@Cu_2_O-Sb_2_O_3_ or Cu_2_O-Sb_2_O_3_ being entirely wrapped up inside the Alg hydrogel. Consequently, the XRD pattern of Alg@Cu_2_O-Sb_2_O_3_ only displayed peaks associated with the Alg phase. Thus, the XRD pattern suggested that Cu_2_O-Sb_2_O_3_ was ensnared inside the Alg hydrogel.

The ATR-FTIR spectrum of Cu_2_O-Sb_2_O_3_ indicated intense bands at 430, 590 and 631 cm^−1^ (Figure 3b). These bands indicate the growth of metal oxide (M=O stretching). The Alg@Cu_2_O-Sb_2_O_3_ nanocatalyst also displayed a high and intense broad band in the same region (400~600 cm^−1^), confirming the presence of Cu_2_O-Sb_2_O_3_ [24,29]. The Alg@Cu_2_O-Sb_2_O_3_ nanocatalyst showed some additional bands at 1032, 1105, 1357, 1461, 1589, 1643, 1725, 2878, and 3200~3400, corresponding to Alg. The ATR-FTIR spectrum of Alg@Cu_2_O-Sb_2_O_3_ indicated C–H and O–H bands at 2878 and 3268 cm^−1^. The symmetric and asymmetric stretching vibration bands at 1461, 1643 and 1725 cm^−1^ were attributed to carboxylate salt ion and C=O. The C–O and C–C–H stretching vibration bands appeared at 1032, 1105 and 1357 cm^−1^. The ATR-FTIR spectra suggest that Cu_2_O-Sb_2_O_3_ and the Alg@Cu_2_O-Sb_2_O_3_ nanocatalyst were prepared without any impurities.

### 3.2. Catalytic Activity of Alg@Cu_2_O-Sb_2_O_3_

#### 3.2.1. Reduction of Organic Dyes

**Figure 4:**
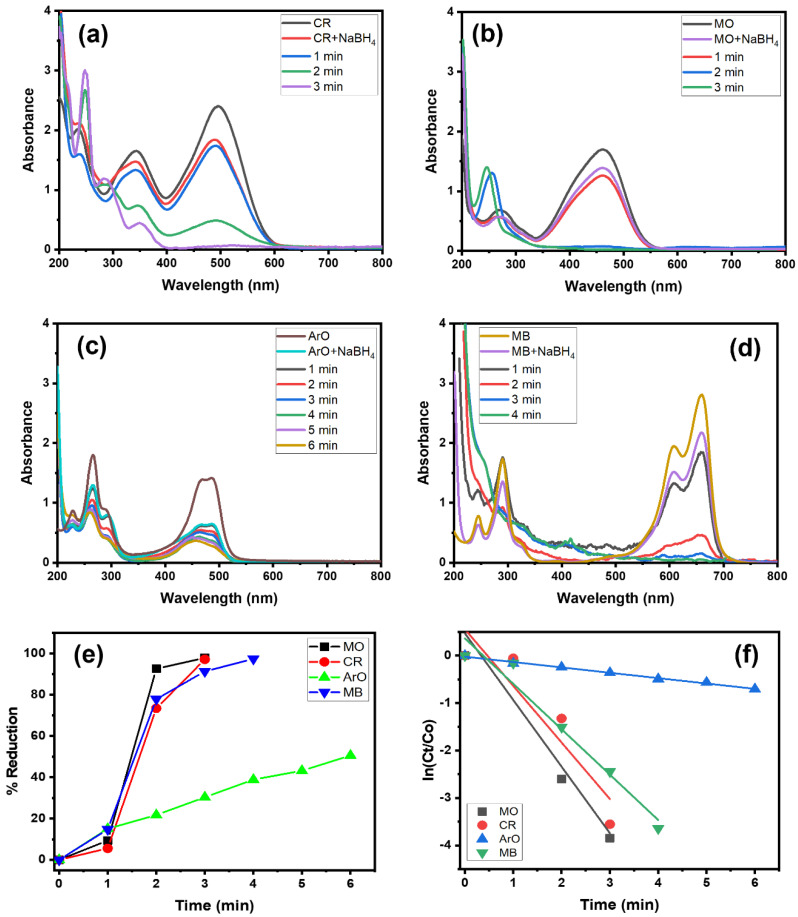
(**a**–**d**) UV–Vis spectra, (**e**) % reduction and (**f**) kinetics of CR, MO, ArO and MB reduction by NaBH_4_ using 10 mg Alg@Cu_2_O-Sb_2_O_3_ nanocatalyst.

Organic dyes are regarded as toxic pollutants, and consequently the prepared Alg@Cu_2_O-Sb_2_O_3_ nanocatalyst was examined in order to evaluate the catalytic activity using NaBH_4_. The Alg@Cu_2_O-Sb_2_O_3_ nanocatalyst was applied for the reduction reaction of CR, MO, ArO and MB. Figure 4a–d exhibits the reduction of CR, MO, ArO and MB by NaBH_4_ and Alg@Cu_2_O-Sb_2_O_3_. The results indicate that Alg@Cu_2_O-Sb_2_O_3_ reduced CR, MO, ArO and MB within 3.0–6.0 min (Figure 4a–d). The current findings suggest the reduction of the azo (-N=N-) group present in dye molecules through the transferring of electrons from BH_4_^−^ to Alg@Cu_2_O-Sb_2_O_3_ and, further, through shifting electrons toward the acceptor dye molecules [30,31]. The conversion (%) was 97.14%, 97.86%, 50.59% and 97.38% for CR, MO, ArO and MB, respectively, within 4.0 min, 3.0 min, 6.0 min and 4.0 min (Figure 4e). The results suggested a high catalytic activity of Alg@Cu_2_O-Sb_2_O_3_ toward the dyes reduction.

The pseudo-first-order equation was utilized to evaluate the reaction kinetics [32].

(2) $$r=\frac{dc}{dt}=ln\frac{c_{t}}{c_{0}}=-k_{app}t$$

where *r* stands for the reactant rate; *C* is the reactant concentration; *t* stands for the time of reaction; *k* represents the reaction rate constant; *C_t_* stands for the dyes concentration at time *t*; and *C*_0_ stands for the dyes concentration at time 0.

The current study proves that the dyes reduction obeys pseudo-first-order kinetics. The rate constants were assessed as being 1.1929 min^−1^, 1.4045 min^−1^, 0.1131 min^−1^ and 0.9571 min^−1^ for CR, MO, ArO and MB, respectively. These high-rate constants suggest that there is a quick diffusion of BH_4_^-^ and dye molecules into Alg@Cu_2_O-Sb_2_O_3_, where electron transfers take place from NaBH_4_ to dye molecules and quickly diffuse out the product.

#### 3.2.2. Reduction of K_3_[Fe(CN)_6_]

**Figure 5:**
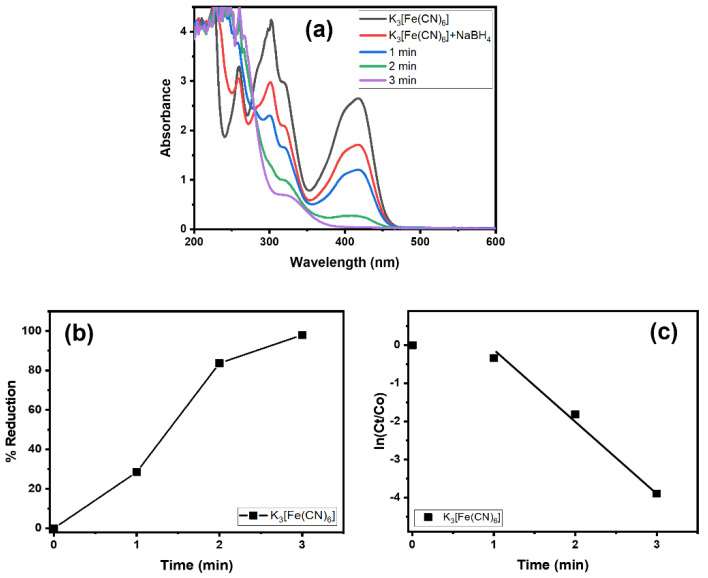
(**a**) UV–vis spectra, (**b**) % reduction and (**c**) kinetics of K_3_[Fe(CN)_6_] in the presence of NaBH_4_ using 10 mg Alg@Cu_2_O-Sb_2_O_3_ nanocatalyst.

Alg@Cu_2_O-Sb_2_O_3_ was tested in the reduction reaction of K_3_[Fe(CN)_6_] and NaBH_4_. At first, the K_3_[Fe(CN)_6_] reduction was only performed by NaBH_4_ without Alg@Cu_2_O-Sb_2_O_3_. The K_3_[Fe(CN)_6_] reduction was very slow without Alg@Cu_2_O-Sb_2_O_3_. Then, 10 mg of Alg@Cu_2_O-Sb_2_O_3_ were placed into the mixture of NaBH_4_ and K_3_[Fe(CN)_6_]. Using visual observation, it was noticed that K_3_[Fe(CN)_6_] bearing a yellow color faded immediately within few seconds and that within 3.0 min the absorbance band at 415 nm declined and disappeared, as demonstrated in Figure 5a. This indicates the reduction of Fe^+3^ to Fe^+2^ and hence the formation of [Fe(CN)_6_]^−4^ [33]. The conversion (%) was 97.98% for K_3_[Fe(CN)_6_] within 3.0 min (Figure 5b), and thus the current study indicated that a good reduction was attained by Alg@Cu_2_O-Sb_2_O_3_; additionally, the results suggested a high catalytic property of Alg@Cu_2_O-Sb_2_O_3_ toward the K_3_[Fe(CN)_6_] reduction. The kinetics of the K_3_[Fe(CN)_6_] reduction were analyzed by applying Equation (2), and the rate constant was assessed to be 1.3180 min^−1^.

#### 3.2.3. Reduction of Nitrophenols

**Figure 6:**
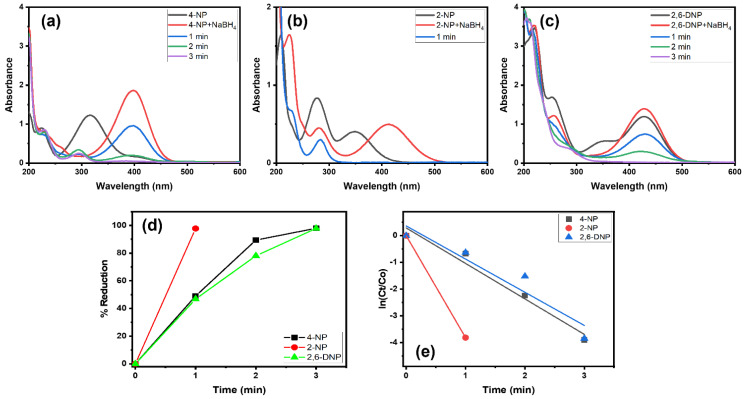
(**a**–**c**) UV–Vis spectra, (**d**) % reduction and (**e**) kinetics of 4-NP, 2-NP and 2,6-DNP in the presence of NaBH_4_ using 10 mg Alg@Cu_2_O-Sb_2_O_3_ nanocatalyst.

Alg@Cu_2_O-Sb_2_O_3_ was also tested in the reduction reaction of 4-NP, 2-NP and 2,6-DNP with NaBH_4_. Figure 6a reveals that 4-NP displayed an absorption peak at 317 nm. The addition of NaBH_4_ caused a shift in absorption from 317 to 400 nm, signifying the formation of 4-nitrophenolate ions [24,34]. After adding Alg@Cu_2_O-Sb_2_O_3_, the 4-nitrophenolate band at 400 nm gradually declined with time, which indicated that the –NO_2_ group present in 4-NP got reduced to the –NH_2_ group. The decline in the 4-nitrophenolate ion peak was faster for Alg@Cu_2_O-Sb_2_O_3_, and the reaction was completed within 3.0 min. Similarly, Alg@Cu_2_O-Sb_2_O_3_ was further analyzed in the reduction reaction of 2-NP and 2,6-DNP with NaBH_4_ using UV–Vis spectrometry. Figure 6b,c illustrates that 2-NP and 2,6-DNP gave absorption peaks at 347 nm and 428 nm. Upon adding NaBH_4_ solution, a change of color was noticed along with a shift in the absorption band from 347 nm to 413 nm for 2-NP, signifying the existence of nitrophenolate ions [24,34]. Furthermore, upon adding Alg@Cu_2_O-Sb_2_O_3_, the absorption peaks recorded at 413 nm and 428 nm declined, while, on the other hand, another absorption peak at 280 nm could be seen with an increasing trend in intensity. The results verify the conversion of nitrophenols to aminophenol by reducing NO_2_ present in 2-NP and 2,6-DNP (Figure 6b,c). The 2-NP and 2,6-DNP reduction reactions were faster in the presence of Alg@Cu_2_O-Sb_2_O_3_, and both reactions were completed within 1.0 min and 3.0 min, respectively. Alg@Cu_2_O-Sb_2_O_3_ reduced more than 97% of 4-NP, 2-NP and 2,6-DNP in 3.0 min, 1.0 min and 3.0 min, respectively. Further, rate constants were obtained by applying Equation (2), and they were 1.3279 min^−1^, 3.8187 min^−1^ and 1.2426 min^−1^ for 4-NP, 2-NP and 2,6-DNP, respectively. These results verify that the Alg@Cu_2_O-Sb_2_O_3_ nanocatalyst is an effective catalyst, displaying more selectivity toward 2-NP.

##### Effect of Catalyst Amount

**Figure 7:**
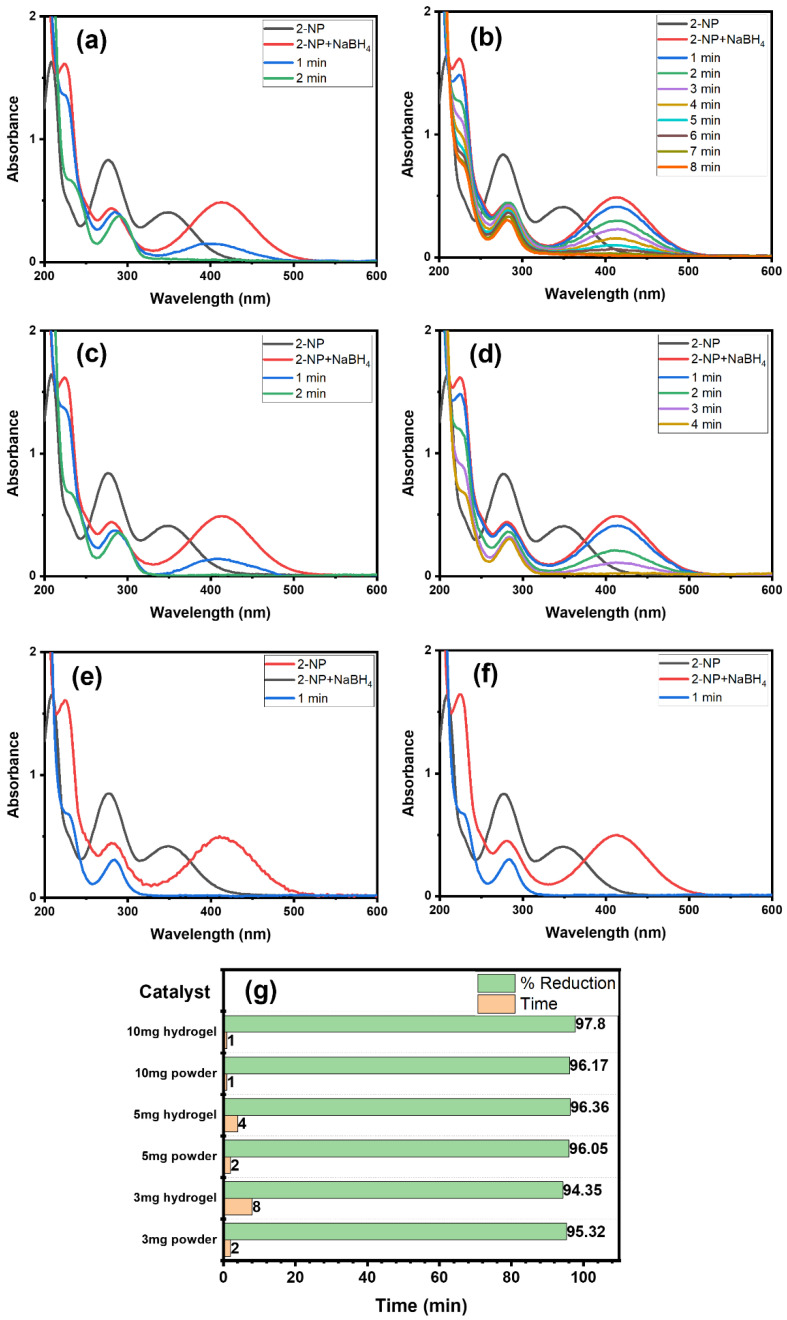
UV–Vis spectra: (**a**) 3 mg Cu_2_O-Sb_2_O_3_, (**b**) 3 mg Alg@Cu_2_O-Sb_2_O_3_, (**c**) 5 mg Cu_2_O-Sb_2_O_3_, (**d**) 5 mg Alg@Cu_2_O-Sb_2_O_3_, (**e**) 10 mg Cu_2_O-Sb_2_O_3_, and (**f**) 10 mg Alg@Cu_2_O-Sb_2_O_3_, (**g**) % reduction with the required time for 2-NP reduction in the presence of NaBH_4_.

For a comprehensive study, we examined the impact of Alg@Cu_2_O-Sb_2_O_3_ hydrogel and powder Cu_2_O-Sb_2_O_3_ amounts on 2-NP reduction using NaBH_4_ reducing agent. Hence, 3, 5 and 10 mg each of Cu_2_O-Sb_2_O_3_ and Alg@Cu_2_O-Sb_2_O_3_ nanocatalysts were added for the reduction of 2-NP to ascertain the impact of catalyst quantity (Figure 7). Figure 7g exhibits the graph of catalyst quantity vs. % reduction as well as the time consumed while maintaining constant amounts of NaBH_4_ and 2-NP. The Alg@Cu_2_O-Sb_2_O_3_ quantity plays a major role in speeding up the reaction and lowering the reaction time. 3 mg, 5 mg and 10 mg of Alg@Cu_2_O-Sb_2_O_3_ reduced 2-NP in 8.0 min, 4.0 min and 1.0 min, respectively. Meanwhile, similar amounts of powder Cu_2_O-Sb_2_O_3_ took 2.0 min and 1.0 min to reduce 2-NP. Thus, the role of Cu_2_O-Sb_2_O_3_ and Alg@Cu_2_O-Sb_2_O_3_ was evidently noticed, and hence, the Cu_2_O-Sb_2_O_3_ and Alg@Cu_2_O-Sb_2_O_3_ quantity plays a substantial role in the rate of 2-NP reduction. Therefore, Cu_2_O-Sb_2_O_3_ is very effective, but powder Cu_2_O-Sb_2_O_3_ has limitations, i.e., it is difficult to separate from the reaction in comparison with the present hydrogel. The powder catalyst has the drawback that it cannot be collected and separated from reaction media in order to recycle it. Hence, a requirement for a good catalyst would be to remove or reduce these concerns. Consequently, Cu_2_O-Sb_2_O_3_ was packed in hydrogel for the purpose of handling and surmounting all the above-mentioned concerns.

##### Effect of NaBH_4_ Concentration

**Figure 8:**
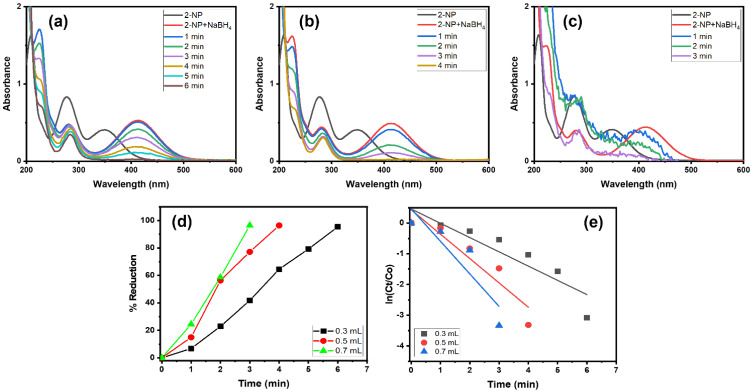
UV–Vis spectra in the presence of (**a**) 0.3 mL, (**b**) 0.5 mL and (**c**) 0.7 mL NaBH_4_, (**d**) % reduction and (**e**) kinetics of 2-NP using 5 mg Alg@Cu_2_O-Sb_2_O_3_ nanocatalyst.

The impact of the NaBH_4_ amount was assessed in the 2-NP reduction using the Alg@Cu_2_O-Sb_2_O_3_ nanocatalyst. To scrutinize various quantities of NaBH_4_, 0.3, 0.5 and 0.7 mL of 0.1 M NaBH_4_ were utilized with 5 mg of Alg@Cu_2_O-Sb_2_O_3_. Figure 8d shows the % reduction against the time plot. 2-NP got reduced up to 95.40%, 96.36% and 96.43% using 0.3, 0.5 and 0.7 mL of 0.1 M NaBH_4_ solution. Further, the reaction rates calculated in the current study were 0.4649 min^−1^, 0.7937 min^−1^ and 1.0599 min^−1^.

##### Effect of 2-NP Concentration

**Figure 9:**
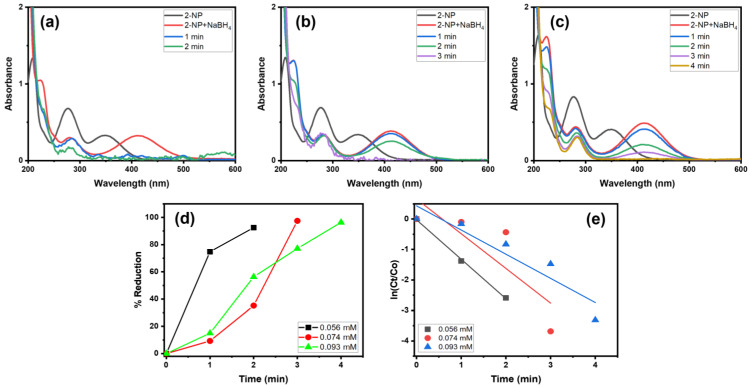
UV–Vis spectra of 2-NP reduction: (**a**) 0.065 mM, (**b**) 0.074 mM, and (**c**) 0.093 mM, (**d**) % reduction and (**e**) kinetics using 5 mg Alg@Cu_2_O-Sb_2_O_3_ and 0.5 mL NaBH_4_.

Moreover, we examined the catalytic behavior of Alg@Cu_2_O-Sb_2_O_3_ in the reduction of different concentrations of 2-NP in the presence of NaBH_4_ (Figure 9). Consequently, various amounts of 2-NP solution (0.056, 0.074 and 0.093 mM) were used to ascertain the catalytic ability of Alg@Cu_2_O-Sb_2_O_3_. Alg@Cu_2_O-Sb_2_O_3_ reduced 92.49%, 97.49% and 97.36% of 0.056, 0.074 and 0.093 mM 2-NP in 2.0, 3.0 and 4.0 min with reaction rates of 1.2946 min^−1^, 1.1388 min^−1^ and 0.7939 min^−1^. Thus, a lower concentration of 2-NP reduces more efficiently when compared to high-concentrated 2-NP.

##### Recyclability of Alg@Cu_2_O-Sb_2_O_3_

**Figure 10:**
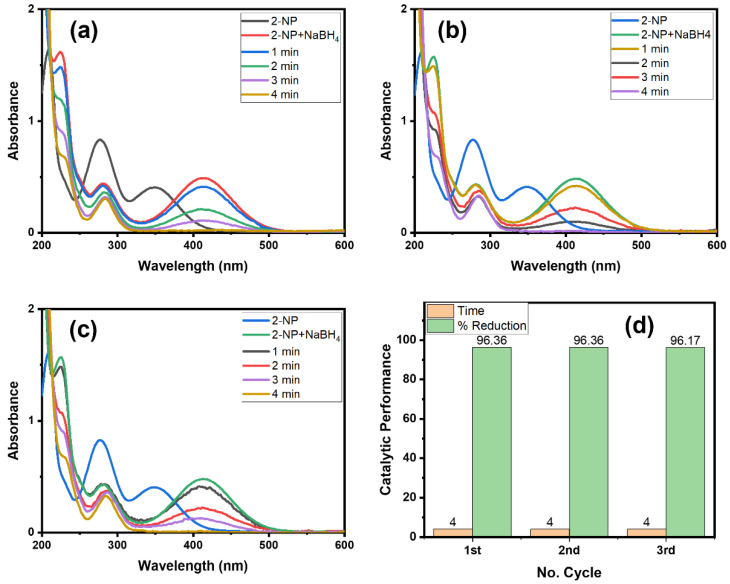
UV–Vis spectra for the reduction of 2-NP: (**a**) 1st cycle, (**b**) 2nd cycle, (**c**) 3rd cycle, and (**d**) comparative reduction time and % reduction in terms of recyclability using 5 mg Alg@Cu_2_O-Sb_2_O_3_ in 2 mL 2-NP and 0.5 mL NaBH_4_.

Catalyst consistency and recovery are important from an environmental and cost-based point of view [13,35]. Therefore, we checked the recyclability property of the Alg@Cu_2_O-Sb_2_O_3_ nanocatalyst (Figure 10). We have analyzed the Alg@Cu_2_O-Sb_2_O_3_ nanocatalyst four times for an easy removal of the product by means of just removing the hydrogel. Figure 10d specifies the time utilized for the reduction reaction of 2-NP in every cycle with the same Alg@Cu_2_O-Sb_2_O_3_ nanocatalyst, which suggests the stability as well as the recyclability property of Alg@Cu_2_O-Sb_2_O_3_. Alg@Cu_2_O-Sb_2_O_3_ efficiently reduced 2-NP in 4.0 min until the 3^rd^ cycle, which suggests that Alg@Cu_2_O-Sb_2_O_3_ can be used numerous times with no loss in catalytic activity. The results were compared with the literature [36,37,38,39,40], and it was fount that Alg@Cu_2_O-Sb_2_O_3_ is an efficient catalyst that can be used several times. This indicate that nanocomposite can play important role as a catalyst since nanocomposites have shown an enormous range of applications in different sectors [41,42,43].

## 4. Conclusions

Cu_2_O-Sb_2_O_3_ nanoparticles were prepared and placed inside the Alg hydrogel. Both Cu_2_O-Sb_2_O_3_ and Alg@Cu_2_O-Sb_2_O_3_ nanocatalysts were excellent catalysts for the selective reduction reaction of 2-NP. The influence of various parameters on the 2-NP reduction using Alg@Cu_2_O-Sb_2_O_3_ showed that increasing the catalyst and NaBH_4_ amounts and decreasing the 2-NP concentration preceded a greater catalytic efficiency. The rate constant when utilizing 10 mg Alg@Cu_2_O-Sb_2_O_3_ was calculated as being 3.8187 min^−1^ for the 2-NP reduction. Lastly, Alg@Cu_2_O-Sb_2_O_3_ maintained a greater activity after recyclability assessments.

## Data Availability

The data presented in this study are available on request from the corresponding author.
